# Distinct patterns of simple sequence repeats and GC distribution in intragenic and intergenic regions of primate genomes

**DOI:** 10.18632/aging.101025

**Published:** 2016-09-16

**Authors:** Wen-Hua Qi, Chao-chao Yan, Wu-Jiao Li, Xue-Mei Jiang, Guang-Zhou Li, Xiu-Yue Zhang, Ting-Zhang Hu, Jing Li, Bi-Song Yue

**Affiliations:** ^1^ Key Laboratory of Bio-resources and Eco-environment (Ministry of Education), College of Life Sciences, Sichuan University, Chengdu 610064, China; ^2^ College of Life Science and Engineering, Chongqing Three Gorges University, Chongqing 404100, China; ^3^ College of Environmental and Chemistry Engineering, Chongqing Three Gorges University, Chongqing 404100, China; ^4^ College of Sport and Health, Chongqing Three Gorges University, Chongqing 404100, China

**Keywords:** simple sequence repeats, GC, genomic regions, patterns, primate genomes

## Abstract

As the first systematic examination of simple sequence repeats (SSRs) and guanine-cytosine (GC) distribution in intragenic and intergenic regions of ten primates, our study showed that SSRs and GC displayed nonrandom distribution for both intragenic and intergenic regions, suggesting that they have potential roles in transcriptional or translational regulation. Our results suggest that the majority of SSRs are distributed in non-coding regions, such as the introns, TEs, and intergenic regions. In these primates, trinucleotide perfect (P) SSRs were the most abundant repeats type in the 5′UTRs and CDSs, whereas, mononucleotide P-SSRs were the most in the intron, 3′UTRs, TEs, and intergenic regions. The GC-contents varied greatly among different intragenic and intergenic regions: 5′UTRs > CDSs > 3′UTRs > TEs > introns > intergenic regions, and high GC-content was frequently distributed in exon-rich regions. Our results also showed that in the same intragenic and intergenic regions, the distribution of GC-contents were great similarity in the different primates. Tri- and hexanucleotide P-SSRs had the most GC-contents in the 5′UTRs and CDSs, whereas mononucleotide P-SSRs had the least GC-contents in the six genomic regions of these primates. The most frequent motifs for different length varied obviously with the different genomic regions.

## INTRODUCTION

Simple sequence repeats (SSRs), or microsatellites, are tandem repeats of 1–6 bp DNA motifs [1], which are distributed in both coding and noncoding regions of eukaryotic and prokaryotic genomes [2,3] and exhibit high degree of polymorphism. Also, some SSRs are preferentially distributed in peri-centromeric heterochromatic regions [4] and were lack of sequence conservation in centromere [5]. SSR loci have a high mutation rate (10^−6^ to 10^−2^/ generation) which changes the number of SSR repeat unit and repeat tracts [6,7].

SSR mutations are more often caused by the number changes of the repeating motifs, not by point mutations [8-10]. The indefinite growth of number of SSR motifs is prevented by the accumulation of base substitutions, and short SSRs should have a lower SSR mutation rate than longer SSRs. Mutation rates of SSR-rich gene are much higher than other parts of the genome [9]. The instability of SSRs is primarily due to slipped-strand mispairing errors during DNA replication [6,11,12]. The majority of slippage insertions/ deletions would be corrected by the mismatch repair system, and only the small part of sites that are not repaired lead to SSR mutations [13].

There are current evidences that SSR expansions or contractions within genome sequences can affect functions of these sequences, even lead to phenotypic changes [14,15]. In 5′-untranslated regions (5′UTRs), SSR expansions and/or contractions can affect gene transcription or regulation, and in protein-coding sequences (CDSs), they can result in the phenotype modification [8,16-18], even lead to the generation of toxic or malfunctioning proteins [19]. For example, the expansion of the (GAG)_n_ motif in the coding region of the Huntington's disease (HD) gene in humans can lead to Huntington's disease [20,21]. SSR variation within intron regions can regulate gene expression, translation, mRNA splicing, and gene silencing [20,22,23], and in 3′-untranslated regions (3′UTRs) they are involved in gene silencing and transcription slippage [20,24]. In addition, the alteration of SSR length within promoter regions may affect transcription factor binding and alter the level and specificity of gene transcription [25].

So far, no systematic research regarding SSRs variation and characterization has been conducted on a genome-wide scale in the primates. The rapid advance of sequencing technologies has made a number of primate genomes available to investigate the characteristics and distributions of SSRs in the intragenic (i.e., 5′UTRs, CDSs, introns, and 3′UTRs) and intergenic regions. The genome sequence data from ten primates: *Otolemur garnetti* (*OtoGar*), *Callithrix jacchus* (*CalJac*), *Macaca mulatta* (*MacMul*), *Chlor*ocebus *sabaeus* (*ChlSab*), *Papio anubis* (*PapAnu*), *Nomascus leucogenys* (*NomLeu*), *Gorilla gorilla* (*GorGor*), *Pongo abelii* (*PonAbe*), *Pan troglodytes* (*PanTro*), *Homo sapiens* (*HomSap*), were used in the study, we detected and characterized SSRs and examined their distributions and variations in intragenic and intergenic regions. Furthermore, we addressed the questions of whether the abundance of different SSR types and motifs are similar or not in different genomic regions and how GC-content of SSR differ in 5′UTRs, CDSs, introns, 3′UTRs, transposable elements (TEs), and intergenic regions. This research will facilitate our understanding of SSRs and their potential biological functions in transcription or translation in the primates.

## RESULTS

### The number and abundance of SSRs in primate genomes

**Figure 1:**
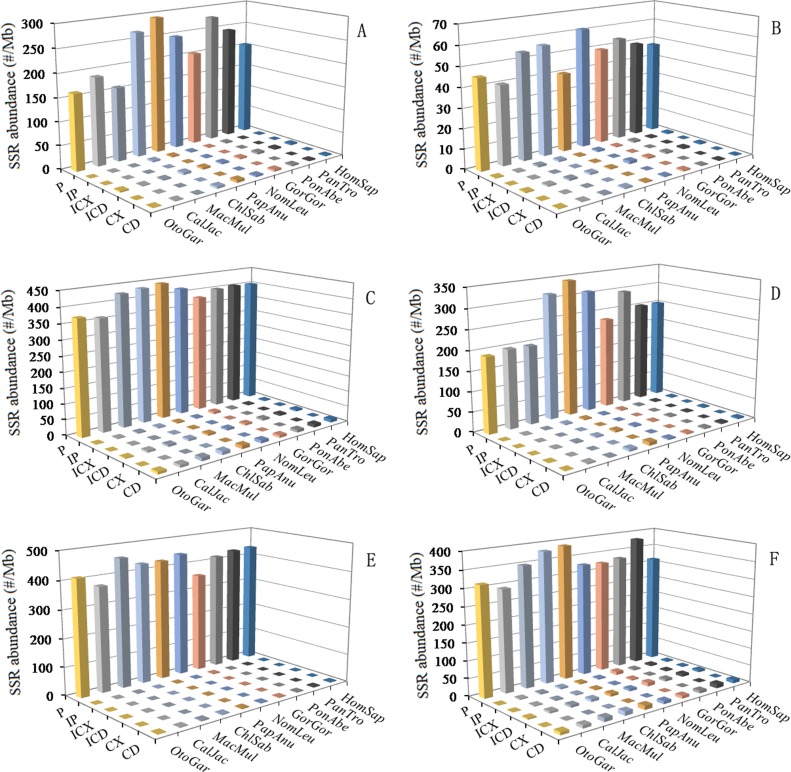
SSRs abundance of six categories in different intragenic and intergenic regions of primates ABCDEF represent 5′UTRs, CDSs, introns, 3′UTRs, TEs, and intergenic regions, respectively.

**Figure 2:**
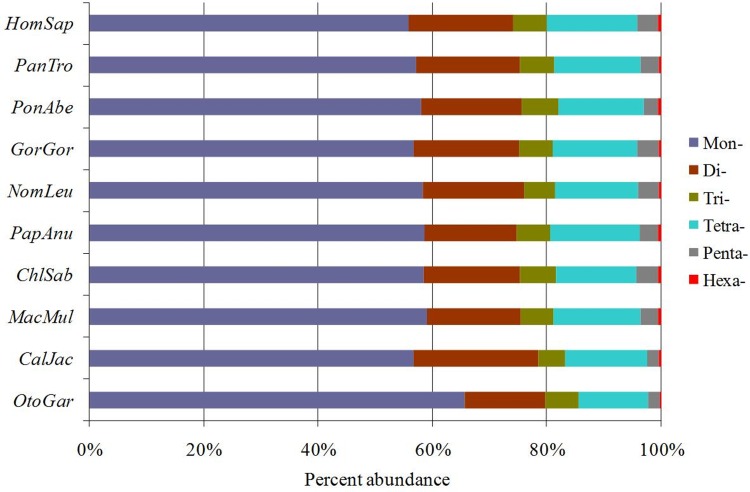
The distribution of SSRs in ten primate genomes Percentages were calculated according to the total number of each SSR category divided by the total number of SSRs for that organism.

The six categories of SSRs were found in each of these primate genomic sequences by using computer software MSDB for a genome-wide scan (Table 1). P-SSRs was the most abundant type, followed by the CD-SSRs and ICD-SSRs, and the least was in the CX-SSRs in these primate species (Table 1). The relative abundances of the same SSR types showed great similarity in the primate species. In the 5′UTRs, CDSs, introns, 3′UTRs, TEs, and intergenic regions of these primates, P-SSRs was the most abundant type, and the least was in the CX-SSRs; the introns and TEs had the most abundant P-SSRs, followed by the pattern: intergenic regions > 5′UTRs > 3′UTRs, and the least was the CDSs (Figure 1). The number and relative abundance of mono- to hexanucleotide P-SSRs across these species genomes are presented in Table 2. Results here indicated that the number and relative abundance of the same repeat type of mono- to hexanucleotides P-SSRs showed great similarity in the ten primate genomes. Mononucleotide P-SSRs were the most abundant category, followed by the pattern: di- > tetra-> tri- > penta- > hexanucleotide P-SSRs (Table 2). The proportion of mono- to hexanucleotide P-SSRs was also very similar in these primate genomes (Figure 2). Mononucleotide P-SSRs were the maximum ratio, accounting for 55.80% ~ 65.62% of all P-SSRs, followed by the pattern: di- > tetra- > tri- > penta- > hexanucleotide P-SSRs. The comparison among the whole genomes of these primates clearly shows that *Otolemur garnetti* has a higher percentage of mononucleotide P-SSRs (65.62%) and *Callithrix jacchus* has a great affinity for dinucleotide repeats (21.76%) compared to other primates. The number of SSRs is closely positive correlated with genome size (Pearson, *r* = 0.742, *p* < 0.05) and relative abundance (Pearson, *r* = 0.685, *p* < 0.05) in these primate genomes. Neither relative abundance nor relative density of SSRs in these primate genomes was significantly correlated with genome size (Pearson, *r* < 0.465, *p* > 0.05).

**Table 1:** Relative abundance of the six categories of SSRs in the primate genomes

Type	*OtoGar*	*CalJac*	*MacMul*	*ChlSab*	*PapAnu*	*NomLeu*	*GorGor*	*PonAbe*	*PanTro*	*HomSap*
CD-SSRs	8.06	8.19	10.93	11.48	11.18	8.28	7.88	7.76	8.73	8.92
CX-SSRs	0.29	0.59	0.94	1.04	0.96	0.50	0.40	0.38	0.49	0.62
ICD-SSRs	3.10	5.51	6.44	7.17	6.84	5.95	8.76	6.45	7.67	5.88
ICX-SSRs	0.61	2.15	2.65	2.71	2.71	2.03	2.99	1.71	2.37	2.05
IP-SSRs	1.14	2.62	3.46	4.54	3.78	3.15	8.61	3.69	3.99	4.07
P-SSRs	349.88	346.57	419.09	446.24	438.66	378.7	356.94	357.45	356.57	381.4

Note: Compound, CD; interrupted compound, ICD; complex, CX; interrupted complex, ICX; Perfect, P;interrupted perfect, IP.

**Table 2:** Number and abundance of mono‐ to hexanucleotide P‐SSRs in the whole genome of ten primates

Repeat type		*OtoGar*	*CalJac*	*MacMul*	*ChlSab*	*PapAnu*	*NomLeu*	*GorGor*	*PonAbe*	*PanTro*	*HomSap*
Mon-	No. of SSRs	578,505	572,440	707,851	716,491	760,821	654,188	588,219	710,219	673,472	660,459
	Abundance (No./Mb)	229.59	196.38	247.18	256.84	258.05	220.86	201.6	206.39	203.49	213.39
Di-	No. of SSRs	123,644	219,782	196,954	206,020	209,660	197,634	191,208	215,893	213,545	216,948
	Abundance (No./Mb)	49.07	75.4	68.78	73.85	71.11	66.72	65.53	62.74	64.52	70.09
Tri-	No. of SSRs	51,335	47,936	69,344	76,567	76,770	60,829	61,069	78,912	70,047	69,569
	Abundance (No./Mb)	20.37	16.44	24.22	27.45	26.04	20.54	20.93	22.93	21.17	22.48
Tetra-	No. of SSRs	107,002	144,273	181,729	173,374	202,358	163,606	154,571	183,841	177,932	186,873
	Abundance (No./Mb)	42.47	49.49	63.46	68.92	66.58	55.23	52.98	53.42	53.76	59.34
Penta-	No. of SSRs	18,843	21,966	38,033	45,949	42,744	40,780	38,248	30,351	37,214	42,805
	Abundance (No./Mb)	7.48	7.54	13.28	16.47	14.5	13.77	14.08	10.02	11.83	13.83
Hexa-	No. of SSRs	2,260	3,835	6,203	7,567	7,023	4,682	5,302	6,707	5,949	7,025
	Abundance (No./Mb)	0.90	1.32	2.17	2.71	2.38	1.58	1.82	1.95	1.8	2.27

### Diversity of P-SSRs in different intragenic and intergenic regions of primates

**Figure 3:**
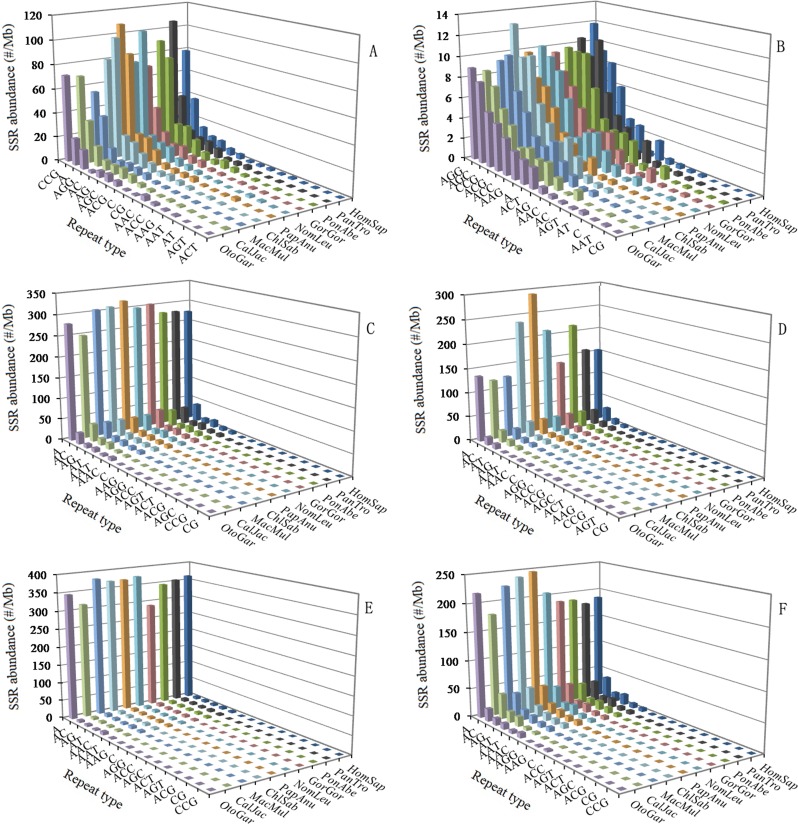
Relative abundance of mono‐ to trinucleo tide P‐SSRs in different intragenic and intergenic regions of ten primates ABCDEF represent 5′UTRs, CDSs, introns, 3′UTRs, TEs, and intergenic regions, respectively.

The abundance of different repeat motifs varied obviously with genomic regions in the ten primates. In the 5′UTRs, the (CCG)_n_ was the most abundant motif, followed by the motif (A)_n_, thirdly the (AGG)_n_, fourthly the (AC)_n_, (AG)_n_, (AGC)_n_, and (ACG)_n_ (Figure 3A). In the CDSs, the (AGC)_n_ and (AGG)_n_ were the most abundant motifs, followed by the motif (CCG)_n_ and (ACG)_n_, thirdly the (A)_n_, (ACC)_n_, (AAG)_n_, and (ACT)_n_, fourthly the (AG)_n_ and (AAC)_n_ (Figure 3B). In the introns, the (A)_n_ was the most abundant motif, followed by the motif (AC)_n_, thirdly the (AG)_n_, (AT)_n_, (AAAT)_n_, and (AAAC)_n_, fourthly the (AAC)_n_, (AAT)_n_, (AAAG)_n_, and (AAGG)_n_, the (CG)_n_ and (CCG)_n_ were relatively infrequent in the intron regions (Figure 3C). In the 3′UTRs, the (A)_n_ was the most abundant motif, followed by the motif (AC)_n_, thirdly the (AT)_n_, fourthly the (AG)_n_, (AAT)_n_, (AAC)_n_, (AAAC)_n_, and (AAAT)_n_ (Figure 3D). In the TEs, the (A)_n_ was the most abundant motif, followed by the motif (AAAT)_n_, thirdly the (AAAC)_n_, fourthly the (AC)_n_, (AG)_n_, (AT)_n_, (AAC)_n_, (AAT)_n_, (AAAG)_n_, and (AAACA)_n_ (Figure 3E). In the intergenic regions, the (A)_n_ was the most abundant motif, followed by the motif (AC)_n_, thirdly the(AG)_n_, (AT)_n_, and (AAAT)_n_, fourthly the (AAC)_n_, (AAT)_n_, (AAAG)_n_, (AAAC)_n_, and (AAGG)_n_ (Figure 3F). Therefore, the motifs of SSRs are not randomly distributed in the 5′UTRs, CDSs, introns, 3′UTRs, TEs, and intergenic regions. There is a noticeable excess of (CCG)_n_ repeats in the 5′UTRs compared to the CDSs, introns, and 3′UTRs, and the (CCG)_n_ repeats was significantly more abundant in the CDSs than that in the introns and 3′UTRs. The (AGG)_n_ and (AGC)_n_ repeats are obvious relatively abundant in 5′UTRs and CDSs compared to other four regions. The (ACG)_n_ repeats are relatively abundant in the 5′UTRs and CDSs compared to other four regions. The (A)_n_ motif was significantly more abundant than the (C)_n_ unit in the six regions. The (AAT)_n_ and (AAC)_n_ motifs are relatively frequent in the introns, 3′UTRs, TEs, and intergenic regions, where their abundance exceeds that of other trinucleotide motifs, and the (CG)_n_ and (CCG)_n_ motifs are relatively infrequent in the four regions.

### Distribution of P-SSRs in different intragenic and intergenic regions of primates

**Figure 4:**
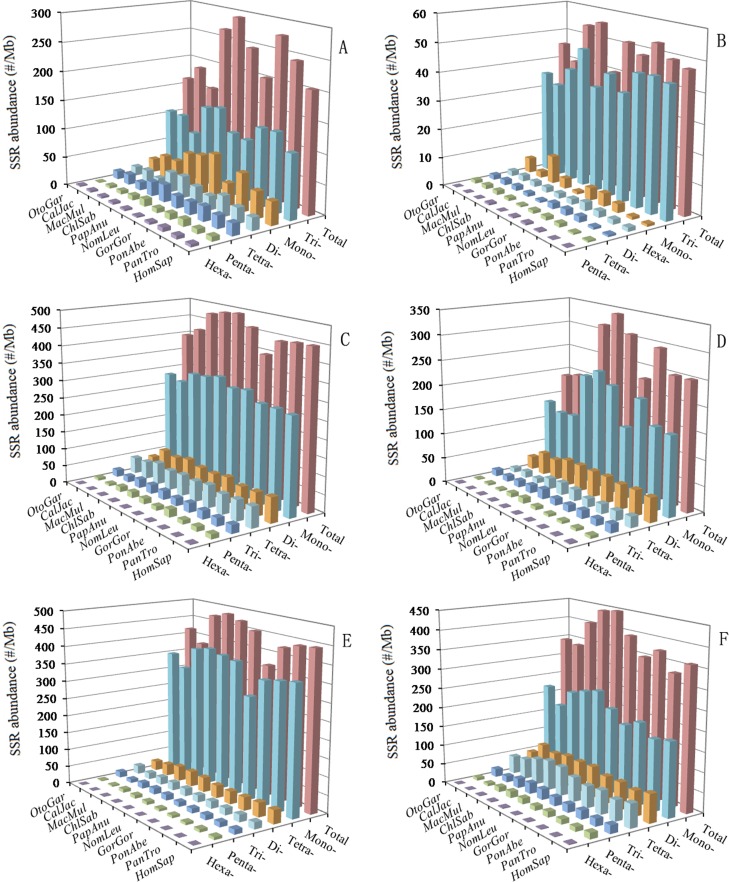
Relative abundance of mono‐ to hexanucleotid e P‐SSRs in different intragenic and intergenic regions of ten primates ABCDEF represent 5′UTRs, CDSs, introns, 3′UTRs, TEs, and intergenic regions, respectively.

In the 5′UTRs, trinucleotide P-SSRs was the most abundant type, followed by the pattern: mono- > di- > tetra- > penta- > hexa-nucleotide P-SSRs in the ten primates (Figure 4A and Supplementary Table 1). In the CDSs, trinucleotide P-SSRs was the most abundant type, followed by the pattern: (1) mono- > hexa- > di- > tetra- > pentanucleotide P-SSRs in the *Otolemur garnettii*, *Callithrix jacchus*, *Macaca mulatta*, *Gorilla gorilla*, *Pongo abelii*, and *Nomascus leucogenys*; (2) hexa- > mono- > di- > tetra- > pentanucleotide P-SSRs in the *Homo sapiens*, *Pan troglodytes*, and *Papio anubis* (Figure 4B and Supplementary Table 2). Tetra- and pentanucleotide P-SSRs, though generally common, were relatively less abundant in the CDSs of these primates. In the introns, mononucleotide P-SSRs was the most abundant type, followed by the pattern: (1) tetra- > di- > tri- > penta- > hexanucleotide P-SSRs in the *Otolemur garnettii*; (2) di- > tetra- > tri- > penta- > hexanucleotide P-SSRs in the remaining nine primates; the least was in the hexanucleotide P-SSRs in these primates (Figure 4C and Supplementary Table 3). In the introns, mono-nucleotide P-SSRs were more than fivefold as frequent as di- and tetranucleotide P-SSRs, and interestingly, the latter are much more frequent than trinucleotide P-SSRs. In the 3′UTRs, mononucleotide P-SSRs was the most abundant type, followed by the pattern: di- > tetra- > tri- > penta- > hexanucleotide P-SSRs (except for *Macaca mulatta* and *Otolemur garnettii*: di- > tri- > tetra- > penta- > hexa-nucleotide P-SSRs) in these primates (Figure 4D and Supplementary Table 4). In the TEs, mononucleotide P-SSRs was the most abundant type, followed by the pattern: tetra- > di- > tri- > penta- > hexanucleotide P-SSRs in the ten primates (Figure 4E and Supplementary Table 5). In the TEs, mononucleotide P-SSRs was more than tenfold as frequent as di- and trinucleotide P-SSRs, and interestingly, the latter are much less frequent than tetranucleotide P-SSRs. In the intergenic regions, mono-mononucleotide P-SSRs was the most abundant type, followed by the pattern: di- > tetra- > tri- > penta- > hexanucleotide P-SSRs in the ten primates (Figure 4F and Supplementary Table 6). Penta- and hexanucleotide P-SSRs were relatively less abundant in the intergenic regions of these primates.

**Table 3:** Overview of the ten primate genomes

Name Parameters	*OtoGar*	*CalJac*	*MacMul*	*ChlSab*	*PapAnu*	*NomLeu*	*GorGor*	*PonAbe*	*PanTro*	*HomSap*
Genome size (Mb)	2,519.72	2,914.96	3,097.37	2,789.64	2,948.38	2,962.06	3,029.54	3,441.23	3,309.56	3,095.09
GC**-**content (in %)	41.11	40.85	40.87	40.81	40.96	40.76	40.54	40.70	40.75	40.91
Number of SSRs	845,502	943,460	1,201,211	1,163,570	1,208,716	1,051,929	1,083,883	1,243,163	1,191,553	1,103,954
Relative abundance (No./Mb)	335.54	323.65	378.81	417.10	409.97	355.13	357.77	361.26	360.03	356.67
Relative density (bp/Mb)	5,903.34	6,624.18	11,020.74	7,440.52	7,684.03	8,440.65	7,321.38	7,287.94	8,784.51	9,122.13
Total length of SSRs (bp)	14,874,768	19,309,199	26,143,832	25,447,470	25,900,059	21,686,338	28,595,581	25,079,455	25,430,842	23,029,134
Genome SSRs content (%^a^)	0.59	0.66	0.84	0.91	0.88	0.73	0.94	0.73	0.77	0.74

a % = total SSRs sequences of whole genome sequnces in the genome level

A comparison among these regions shows that relative abundances and percentage of most of the same mono- to hexanucleotide P-SSRs showed great similarity in the same genomic regions of these primates. Remarkably, the total SSR abundance among all regions for these primates is the most for the introns (Figure 4). There are more than sevenfold difference between the total SSR abundance of the CDSs and introns. These results here indicated that SSRs are more abundant in non-coding regions than coding regions in these primates and that SSR abundances are greater in the introns, TEs, intergenic regions than their whole genomes.

### The GC content of all P-SSRs in the primate genomes

**Figure 5:**
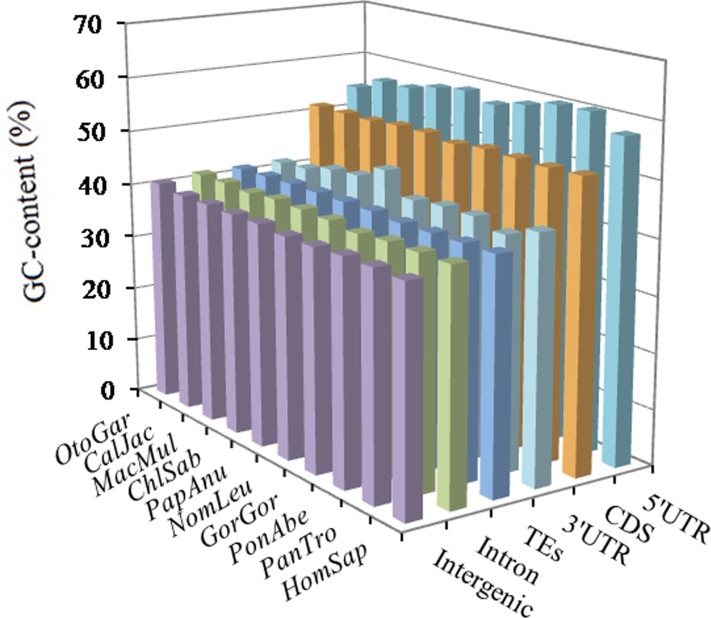
GC‐content of different intragenic and intergenic regions in ten primates

The GC-contents varied greatly among different intragenic and intergenic regions, but, in same intragenic and intergenic regions, the distribution of the GC-content is great similarity. From the results (Figure 5) we can know that 5′UTRs had the most GC-content (ranging 54.39~60.44%), followed by the pattern: CDSs (51.49 ~ 52.14%) > 3′UTRs (41.54 ~ 46.37%) > TEs (41.40 ~ 41.80%) > introns (40.31 ~ 41.61%) > inter-genic regions (39.85 ~ 40.82%). The distribution patterns of AT-contents were great similarity in the same genomic regions of these primates (Supplemen-tary Table 7). From this we can know, high GC-content was distributed in exon-rich regions more frequently than other regions, and the GC-content was not evenly distributed in different genomic regions.

**Figure 6:**
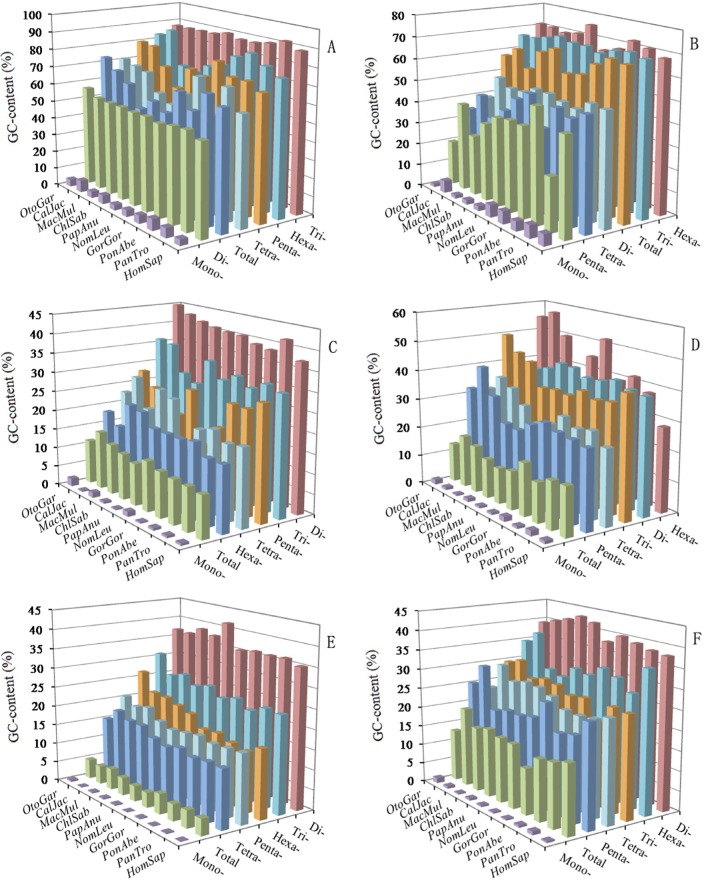
GC‐content of mono‐ to hexanucleotideP‐SSRs in different intragenic and intergenic regions of ten primates ABCDEF represent 5′UTRs, CDSs, introns, 3′UTRs, TEs, and intergenic regions, respectively.

The AT- and GC-content of mono- to hexanucleotide P-SSRs were calculated in the 5′UTRs, introns, CDSs, 3′UTRs, TEs, and intergenic regions of ten primate genomes, which the results were shown in Figure 6 and Supplementary Table 8-13. In the six regions, mononucleotide P-SSRs had the least GC-contents and were significantly less than their total GC-contents in these primate genomes. In the 5′UTRs, we can know that except for the mononucleotide P-SSRs, the GC-content of the remaining nucleotide repeat types are more than their AT-content (Figure 6A and Supplemen-tary Table 8). Trinucleotide P-SSRs had the most GC-content (over 87.40%), followed by the pattern: hexa- > penta- > tetra- > dinucleotide P-SSRs in the 5′UTRs of these primates (Figure 6A). In contrast, the GC-content in dinucleotide P-SSRs were significantly lower than their total GC-content, whereas the GC-content in the tri-, penta- and hexa-nucleotide P-SSRs were more than their total GC-content in the 5′UTRs of these primates (Figure 6A). In the CDSs, the most GC-contents were in tri- and hexanucleotide P-SSRs, ranging from 65.55% (*Nomascus leucogenys*) to 75.63% (*Papio anubis*), which were more than their AT-content, and the GC-content of the remaining nucleotide repeat types were significantly lower than their total GC-content (54.60 ~ 68.79%) in these primates, especially in mononucleotide P-SSRs (Figure 6B and Supplementary Table 9). In the 3′UTRs, except for the hexanucleotide P-SSRs, the GC-content of the remaining nucleotide repeat types were less than their AT-content, and tetra- and pentanucleotide P-SSRs had the second least GC-content (Figure 6D and Supplementary Table 11). In the introns, TEs, and intergenoic regions, we can know that the GC-contents of mono- to hexanucleotide P-SSRs are less than their AT-content, and the most GC-contents were all in dinucleotide P-SSRs in these primates (Figure 6C, E, F and Supplementary Table 10, 12-13). In the introns and TEs, trinucleotide P-SSRs had the second most GC-contents, which were more than GC-contents of tetra-, penta-, and hexanucleotide P-SSRs in the primates. In the TEs, tetra, penta-, and hexa-nucleotide P-SSRs are of similar GC-contents in the primates. In the intergenic regions, hexanucleotide P-SSRs had the second most GC-contents, tri-, tetra, and pentanucleotide P-SSRs were of similar GC-contents, which were less than that of hexanucleotide P-SSRs (Figure 6F and Supplementary Table 13). In contrast, the GC-content in the di- to hexanucleotide P-SSRs were more than their total GC-content in the 3′UTRs, introns, TEs, intergenic regions. In the 3′UTRs, introns, TEs, and intergenoic regions, the total AT-contents ranged from 82.17 % to 93.19%, were significantly higher than their total GC-content; whereas, in the 5′UTRs and CDSs, the total GC-contents ranged from 50.34 % to 73.37%, were significantly higher than their total AT-content in the primates. Therefore, the GC-content of P-SSRs is probably high in exon-rich regions, whereas, the AT-content of P-SSRs is probably quite high in non-coding regions of primates.

## DISCUSSION

In a genome-wide study of SSRs using 10 primate species, there were clearly similarity patterns of SSRs distribution in the primate genomes. Mononucleotides SSRs were the most prevalent repeat type, accounting for 55.80% ~ 65.62% of all SSRs, followed by the pattern: di- > tetra-> tri- > penta- > hexanucleotide SSRs in the study. In the bovid genomes, mononucleotides SSRs were also the most abundant repeat type, accounting for 43.01% – 45.33% of all SSRs, followed by the pattern: di- > tri- > Penta- > tetra- > hexanucleotides SSRs. It has been reported that the abundance of mononucleotide SSRs are more than other nucleotide SSRs in eukaryotic genomes [26,27]. Also, mononucleotide SSRs are the most abundance in all human chromosomes [28]. In contrast, trinucleotide P-SSRs were less abundant than tetranucleotide P-SSRs, and hexanucleotide P-SSRs was the least in the primate genomes. The presence of abundant di- and tetranucleotide SSRs with their features of higher replication slippage than trinucleotide SSRs, especially in the upstream regulatory regions, introns and intergenic regions might be contributing to their high polymorphic potential [29]. Mayer et al. (2010) detected that there was weak correlation between the genome sizes and SSR densities [30]. In 257 virus genomes, the relative SSR densities (bp/kb) showed quite weak correlation with genome size [31]. Our analysis showed that the number of SSRs was significantly correlated with genome size (Pearson, *r* = 0.742, *p* < 0.05) and relative abundance (Pearson, *r* = 0.685, *p* < 0.05) in the primate genomes, suggesting that SSRs might have not contributed significantly to the genome size expansion in evolution. The change of SSR density was consistent with the variations of SSR abundance in the different regions of primates. This will definitely help us improve our understanding of the evolution of SSRs and their roles in gene expression regulation.

### Similarity and diversity of SSR motifs in different genomic regions

The major motifs of mono- to hexanucleotide P-SSR types showed great similarity in the primate whole genomes. We can always find (A/T)-rich motifs among the most common repeat types, such as (AX)_n_, (AAX)_n_, (AAAX)_n_, (AAAAX)_n_, (AAAAAX)_n_ motifs, where X denotes any base other than A, are very abundant in these primate genomes. It has been demonstrated that the (AAAX)_n_ motifs were very abundant in primates and rodents [2]. In the tetranucleotide motifs, the (AAAT)_n_ repeats are the most abundant motifs, followed by the motif (AAAC)_n_, thirdly the (AAAG)_n_, fourthly the (AAGG)_n_ in the primate genomes, this is consistent with previous report [2]. The motifs of mono- to hexanucleotide P-SSR types showed distinct distribu-tion patterns in the intragenic and intergenic regions of primates. Our results showed that the abundance of SSRs are much higher in the introns, TEs, and intergenic regions compared to the other genomic regions. In 42 prokaryotic genomes, the SSR distributions in CDSs were biased toward CDS termini, yielding U-shape SSR abundance curves across the span of the CDSs [32]. In the study, there is also a noticeable excess of (AGC)_n_ and (AGG)_n_ repeats, and (CCG)_n_ constitutes the second most frequent motif in the CDSs compared to the other genomic regions in the primates. The (CG)_n_ are relatively frequent in the 5′UTRs, whereas their abundance are very little in the CDSs, introns, 3′UTRs, TEs, and intergenic regions of the primates. The (CCG)_n_ motifs are relatively infrequent in the introns, TEs, and intergenic regions, where their abundance were less than that of other trinucleotide motifs in the primates. The (CCG)_n_ motifs are the most abundant repeats in 5′UTRs of these primates, whereas (AG)_n_ and (AAG)_n_ were the top-ranked SSR motifs in 5′UTRs of dicots [33]. The (A)_n_ repeats are the most abundant motifs in the introns, 3′UTRs, TEs, and intergenic regons of these primates, rather than (AAT)_n_ repeats, this is inconsistent with previous report [2]. The second most frequent motifs are dinucleotide (AC)_n_ repeats in introns, 3′UTRs, and intergenic regions of these primates might suggest that the motifs may be involved in exon splicing or alternative splicing [10]. (AAC)_n_ and (AAT)_n_ repeats are relatively frequent in introns, TEs, and intergenic regions of these primates, where their occurrence exceeds that of other trinucleotide repeats. We have demonstrated that the (ACG)_n_ and (CCG)_n_ repeats were absolutely presented in these primates, this is inconsistent with previous report [2]. It has been reported that the (CCG)_n_ motifs were predominantly presented in the upstream regions of the genes [34]. Thus, we speculate that the (CCG)_n_ motifs play significant roles in the regulation of gene expression.

Longer repeat units possessed more kinds of motif types than short repeat units. In terms of motif types, hexanucleotide SSRs has the most kinds of motif types, followed by the pattern: penta- > tetra- > tri- > di- > mononucleotide motif types. In our study, mono-nucleotide SSRs has only two kinds of motifs, whereas hexanucleotide motifs has more than 200 kinds of units in these primate genomes: 205 in *Otolemur garnetti*, 211 in *Callithrix jacchus*, 233 in *Gorilla gorilla*, 234 in *Macaca mulatta*, 233 in *Papio anubis*, 237 in *Chlorocebus sabaeus*, 218 in *Pan troglodytes*, 234 in *Pongo abelii*, 211 in *Nomascus leucogenys*, 230 in *homo sapiens*. It was presumed that SSR motifs were not generated randomly in the genomes and motif types may play important roles in gene expression and regulation. In humans, the number variation of repeat units are related to some serious diseases or defects, such as fragile X syndrome [35], spinobulbar muscular atrophy [42], and Huntington's disease [36]. In *Arabidopsis thaliana*, the well-known Bur-0 IIL1 defect generates a detrimental phenotype, which is caused by the expansion of (AAG)_n_ motif in the intron of IIL1 gene [37].

### The variation of SSR abundance in different intragenic and intergenic regions

The abundance of SSRs varies widely between genomes [2,7], and recent evidence suggests a non-random genomic distribution. It has been demonstrated that SSRs in different genomic regions might play different functional roles. For example, SSR expansions or contractions in coding regions can determine whether a gene becomes activated; intronic SSRs can affect gene transcription, mRNA splicing and gene silencing; SSR variations in 5′UTRs could regulate gene expression and SSR expansions in 3′UTRs may cause transcription slippage [20]. It has been reported that changes of SSRs are involved in several human diseases [38-40]. Our results showed that the abundance of different SSR types varies with the genomic region. SSRs have been shown to be more abundant in non-coding regions than in coding regions [2,7,24,41]. In the different genomic regions of the same primates, the introns and TEs had the most abundant P-SSRs, followed by the pattern: intergenic regions > 5′UTRs > 3′UTRs > CDSs. P-SSR abundance is the least in the CDSs, indicating that low SSR abundance may decrease the evaluability of proteins. This may be related to the fact that SSR births/deaths were strongly selected against in CDSs [42].

This evidence has been demonstrated that the mutations of CDSs could cause protein functional changes, loss of function, and protein truncation [20]. In different repeat type of these primates, trinucleotide P-SSRs was the most abundant type in the 5′UTRs and CDSs, whereas mononucleotide P-SSRs was the most abundant type in the 3′UTRs, introns, TEs, and intergenic regions; pentanucleotide P-SSRs was the least in the CDSs, whereas hexanucleotide P-SSRs was the least in the 5′UTRs, introns, 3′UTRs, TEs, and intergenic regions. Trinucleotide SSRs are the most abundant type in the protein-coding regions of all taxa [4]. In the exon regions, in the *Otolemur garnetti* trinucleotide P-SSRs were the most abundant, followed by the pattern: (1) mono- > di- > tetra- > hexa- > pentanucleotide P-SSRs; in the remaining primates mononucleotide P-SSRs were the most abundant, followed by the pattern: tri- > di- > tetranucleotide, and the least was in the penta- and hexanucleotide SSRs. It has been showed that SSRs are significantly enriched within 5′UTRs and their immediate upstream intergenic regions in *Arabidopsis thaliana* and *Oryza sativa* [24,43,44], which belong to the promoter regions where core promoter elements are often represented [45]. In the introns of these primates, the rarity of trinucleotide P-SSRs was also quite pronounced in comparison to di- and tetranucleotide P-SSRs. And we found that the introns didn′t contain more hexanucleotide P-SSRs than exons, which was inconsistent with previous reports [2]. It has been reported that CDSs are preferentially selected with tri- and hexanucleotide SSR motifs [3, 28,30,43,46], which can reduce potential translational frameshift mutations [47]. This evidence can help to explain why tri-fold nucleotide SSR motifs are more frequent in CDSs than other genomic regions. Furthermore, there is strong evolutionary pressure against SSRs expansion in CDSs, which can maintain the stability of the protein products [48].

### The distributional difference of GC-content in different genomic regions of primates

Here, we further examined the nucleotide components in different genomic regions of ten primates. The GC-contents of ten primate genomes showed a remarkably consistent, but GC-contents varied greatly among different intragenic and intergenic regions. In different genomic regions of the primates, the distribution patterns of the GC-content were as followed: 5′UTRs > CDSs > 3′UTRs > TEs > introns > intergenic regions. Thus we can know that high GC-content was frequently distributed in exon-rich regions, and the distribution of GC-content was uneven in the primate genomes. Extreme heterogeneity of local GC-content is one of the most recognizable characteristics in the human genome [49,50]. In rice, the GC-content ranking was 5′UTRs (55.7%) > exons (53.2%) > introns (43.8%) > 3′UTRs (40.2%), whereas, in *Arabidopsis* the GC-content ranking was exons (44.2%) > 5′UTRs (38.3%) > 3′UTRs (33.8%) > introns (32.5%) [24]. Typically, the 5′-ends of a Gramineae gene were up to 25% more rich in GC-content than their 3′-ends [51]. Different classes of TEs tend to have bias for either GC-rich or GC-poor regions [52]. Ancestral Alu sequences have a high GC and CpG content [53,54]. In the study, the motifs of GC-richness were present in the 5′UTRs and CDSs, in which the GC-content were much higher than other genomic regions (Figure 5); whereas the motifs of AT-richness were present in the introns, 3′UTRs, TEs, and intergenic regions, in which the AT-content were much higher than the 5′UTRs and CDSs (Supplementary Table 8-13). It is clear that the top SSR motifs have a strong positively relationship with the GC- or AT-content in different genomic regions. This similar relationship also has been demonstrated in recent years [55]. Therefore, if there is high GC-content in a genomic regions, then the most frequent SSR motifs prefer to be GC-rich instead of AT-rich, and vice versa.

In contrast, the gradient of average GC-content decreases from the 5′UTRs to intron regions by several percent to approximately16.85% in these different genomic regions of the primates. It has been reported that there is a gradient in the GC-content of Gramineae genes, but not eudicot genes [51]. It is an unresolved problem that how GC-content heterogeneities arise in the genome and no model predicts a gradient of GC-content. The GC-content gradients always decreases from 5′- to 3′-ends, which was consistent with the strict directionality of the transcription-related gradient. It may be that there is a gradient effect in the 5′UTR regions because they are also transcribed. The best evidence for a translation-related selection is the sharp transition in GC-content at the start of 5′UTR regions. This makes sense if, in addition to the mutational biases, the (G/C or GC)_n_ repeats are selected to insert in the 5′UTR and CDS regions, and the adjacent noncoding sequences, 3′UTRs and introns, are inherited along with them. It has been speculated on the molecular mechanisms that it would include elements of transcription-coupled DNA repair [56,57], coupled to the process of transcription initiation, elongation, and termination [58]. The overall preference toward higher GC-content is attributable to the low-fidelity polymerases that facilitate replicative bypass [59]. A gradient of GC-content would arise when the repair process aborts or bypasses the lesions to be repaired more frequently than transcription itself [51]. It has been reported that the substitution rates of GC pair strongly negative correlated with the GC-content and exon density [52]. Also, the substitution of telomere surrounding sequences is help to increase the GC-content of their sequences that are within 10-15 Mbp away from the telomere [52]. Births/deaths of SSRs occurred in genomic regions with high substitution rates, protomicrosatellite content, and L1(TE) density, but low GC- and Alu-content [42]. Low GC-content and an abundance of protomicrosatellites facilitate SSRs births/deaths, likely because such sequences have high rates of slippage [60] and substitution [52]. GC-rich Alus have a negative association with SSR births/deaths [42]. Thus, GC- and Alu-content were negative predictors of SSR births/deaths.

## MATERIALS AND METHODS

### Genome sequences and SSR identification

We selected whole genome sequences of ten primates as samples to analyze the SSR distributions in the genomic level. All the genome sequences were downloaded in FASTA format from the Ensembl (http://asia.ensembl.org/index.html). The species, genome size, GC-content, etc., have been summarized in Table 3. The genome size ranged from ~2519.72 Mb (*Otolemur garnetti*) to 3441.23 Mb (*Pongo abelii*). The sequences of the gene models, 5′UTRs, CDSs, introns, 3′UTRs, TEs, and intergenic regions were generated according to the positions in the genome annotations. The intergenic regions referred to the interval sequences between gene and gene that were not included the introns, CDSs, UTRs, and other sequences. SSRs can be grouped into six categories [26,61,62], which were identified and scanned for SSRs of 1-6 bp using the software MSDB (Microsatellite Search and Building Database) downloaded at https://code.google.com/p/msdb/ [63]. To compare our results, we performed a similar analysis of these primate genomes using the same bioinformatics tool and search parameters.

Since primate species are very large genomes, relatively systemic search criteria were adopted in the study, and the parameters for minimum repeat numbers were set as 12, 7, 5, 4, 4, 4 for mono-, di-, tri-, tetra-, penta-, and hexa-nucleotide SSRs, respectively [29]. In this study, repeats with unit patterns being circular permutations and/or reverse complements of each other were grouped together as one type for statistical analysis [64,65]. For tetra- and hexanucleotide repeats, combinations representing perfect di- and tri-nucleotide repeats were filtered from the final counts [26]. The combinations of SSRs for this study will help to a better knowledge of total SSRs occurrence, and their genomic locations will be very useful in selecting SSRs representative of similar repeat classes from different genomic regions as potential markers. To facilitate the comparison among different repeat categories or motifs, we determined relative abundance, which means the number of SSRs per Mb of the sequence analyzed, and relative density, which means the length (in bp) of SSRs per Mb of the sequence analyzed [63,66]. These total numbers have been normalized as relative abundance to allow comparison in the different genomic regions. In the four DNA bases, percentage of guanine (G) plus cytosine (C) was called GC-content in the analyzed sequence.

### Statistical analysis

All data analyses were performed using SPSS version 18.0 and followed standard procedures. The Pearson test was used to reveal the correlation between two variables, including number of SSRs, relative abundance, relative density, genome size, and GC-content, chromosome sequence size.

## SUPPLEMENTARY MATERIAL TABLES



## Abbreviations

AT-content: adenine-thymine content
GC-content: guanine-cytosine content
MSDB: Microsatellite search and building database
SSRs: Simple sequence repeats
P-SSRs: Perfect SSRs
IP-SSRs: Interrupted perfect SSRs
C-SSRs: Compound SSRs
IC-SSRs: Interrupted compound SSRs
CX-SSRs: Complex SSRs
ICX-SSRs: Interrupted complex SSRs
