# Supplementary material for: Distinct patterns of simple sequence repeats and GC distribution in intragenic and intergenic regions of primate genomes
Source: Aging (Albany NY). 2016 Sep 16;8(11):2635–50. doi: 10.18632/aging.101025 (PMC5191860; doi:10.18632/aging.101025)
Supplement: Supplementary file 1 [file aging-08-2635-s001.pdf]

SUPPLEMENTARY MATERIAL

Supplementary Table 1. Percentage of mono- to hexanucleotide P-SSRs in the 5'UTRs of the primates genomes

5'UTR	*OtoGar*	*CalJac*	*MacMul*	*ChlSab*	*PapAnu*	*NomLeu*	*GorGor*	*PonAbe*	*PanTro*	*HomSap*
Size (Mb)	1.95	7.40	4.48	2.91	3.02	3.46	3.20	3.30	3.19	22.28
Mono-	13.92	20.19	24.29	28.69	27.79	28.63	18.80	27.04	18.77	19.43
Di-	11.39	9.63	9.18	11.59	11.57	10.74	11.86	13.42	10.93	10.68
Tri-	63.29	56.54	51.98	47.31	44.83	49.26	51.46	44.30	55.53	53.61
Tetra-	8.23	9.34	8.62	8.31	10.23	7.68	10.79	8.48	9.00	10.11
Penta-	1.90	2.51	3.81	2.81	4.55	2.84	5.08	4.04	3.34	3.92
Hexa-	1.27	1.80	2.12	1.29	1.03	0.84	2.00	2.72	2.44	2.26
Total	100.00	100.00	100.00	100.00	100.00	100.00	100.00	100.00	100.00	100.00

Supplementary Table 2. Percentage of mono- to hexanucleotide P-SSRs in the CDSs of the primates genome

Type	*OtoGar*	*CalJac*	*MacMul*	*ChlSab*	*PapAnu*	*NomLeu*	*GorGor*	*PonAbe*	*PanTro*	*HomSap*
size (Mb)	32.52	67.16	52.61	31.55	35.19	32.66	44.33	32.42	31.64	117.84
Mono-	11.37	5.37	17.53	6.34	2.27	8.27	9.26	5.66	1.99	1.77
Di-	3.65	4.56	3.09	2.21	1.91	3.87	4.44	3.64	1.67	1.47
Tri-	78.15	81.15	73.01	85.22	89.72	81.25	76.34	83.54	91.53	92.58
Tetra-	2.57	3.44	2.64	1.76	1.49	2.02	2.57	2.37	1.22	1.16
Penta-	0.27	0.37	0.39	1.25	0.28	0.95	1.40	0.75	0.26	0.20
Hexa-	3.99	5.11	3.34	3.23	4.32	3.63	5.98	4.04	3.34	2.82
Total	100.00	100.00	100.00	100.00	100.00	100.00	100.00	100.00	100.00	100.00

Supplementary Table 3. Percentage of mono- to hexanucleotide P-SSRs in the introns of the primates genome

Type	*OtoGar*	*CalJac*	*MacMul*	*ChlSab*	*PapAnu*	*NomLeu*	*GorGor*	*PonAbe*	*PanTro*	*HomSap*
size (Mb)	689.23	1310.05	1083.36	783.89	904.98	938.95	958.15	962.14	959.90	4424.67
Mono-	71.64	65.00	64.42	63.74	65.22	65.06	61.86	63.88	63.05	61.84
Di-	9.73	17.29	14.12	15.76	13.95	14.60	14.96	14.79	15.09	15.97
Tri-	5.00	4.08	5.13	5.20	5.13	4.79	5.31	5.45	5.19	5.36
Tetra-	11.54	11.71	12.82	11.30	12.08	12.57	12.94	12.81	13.30	13.34
Penta-	1.87	1.60	3.04	3.44	3.14	2.60	4.47	2.61	2.93	2.95
Hexa-	0.23	0.33	0.47	0.56	0.48	0.37	0.47	0.45	0.43	0.54
Total	100.00	100.00	100.00	100.00	100	100.00	100.00	100.00	100.00	100.00

Supplementary Table 4. Percentage of mono- to hexanucleotide P-SSRs in the 3'UTRs of the primates genome

Type	*OtoGar*	*CalJac*	*MacMul*	*ChlSab*	*PapAnu*	*NomLeu*	*GorGor*	*PonAbe*	*PanTro*	*HomSap*
Size (Mb)	11.47	21.75	13.56	16.71	12.04	20.34	16.12	17.38	15.56	79.72
Mono-	73.12	62.89	64.88	69.58	69.15	69.30	61.45	69.55	64.36	62.28
Di-	13.62	22.36	20.89	14.84	12.15	16.54	22.63	15.55	20.01	19.22
Tri-	6.81	5.81	6.72	4.54	4.67	4.29	5.93	5.47	5.32	6.81
Tetra-	4.66	6.46	4.96	8.20	11.13	7.32	7.34	7.36	7.62	8.67
Penta-	1.08	2.14	1.84	2.39	2.54	2.15	2.17	1.69	2.10	2.22
Hexa-	0.72	0.35	0.71	0.45	0.37	0.40	0.49	0.39	0.59	0.79
Total	100.00	100.00	100.00	100.00	100.00	100.00	100.00	100.00	100.00	100.00

Supplementary Table 5. Percentage of mono- to hexanucleotide P-SSRs in the TEs of the primates genome

Type	*OtoGar*	*CalJac*	*MacMul*	*ChlSab*	*PapAnu*	*NomLeu*	*GorGor*	*PonAbe*	*PanTro*	*HomSap*
Size (Mb)	905.63	1257.35	1344.54	1304.33	1331.08	1321.23	1190.72	1365.45	1359.57	1367.36
Mono-	83.53	82.88	80.94	80.93	81.11	83.22	78.96	81.41	80.86	81.82
Di-	5.62	4.64	4.55	4.55	4.54	4.33	5.59	4.69	4.70	4.36
Tri-	3.87	2.63	3.47	3.48	3.42	2.91	3.57	3.28	3.16	3.07
Tetra-	6.30	8.64	8.86	8.88	8.81	7.86	9.75	8.79	9.20	8.78
Penta-	0.64	1.12	1.96	1.93	1.91	1.52	1.93	1.66	1.88	1.77
Hexa-	0.03	0.09	0.22	0.22	0.22	0.16	0.20	0.18	0.20	0.20
Total	100.00	100	100.00	100.00	100.00	100.00	100.00	100.00	100.00	100.00

Supplementary Table 6. Percentage of mono- to hexanucleotide P-SSRs in the intergenic regions of the primates genome

Type	*OtoGar*	*CalJac*	*MacMul*	*ChlSab*	*PapAnu*	*NomLeu*	*GorGor*	*PonAbe*	*PanTro*	*HomSap*
size (Mb)	1718.68	1770.85	1913.80	1933.97	1936.47	1781.82	1874.75	2090.02	2213.39	1733.56
Mono-	64.41	53.56	56.13	54.35	56.24	55.10	53.97	55.38	54.35	52.11
Di-	13.98	23.94	17.74	17.81	17.25	20.07	19.68	18.62	19.57	20.17
Tri-	5.97	4.76	5.96	6.32	6.07	5.70	5.96	6.00	5.99	5.90
Tetra-	13.14	15.35	16.38	17.16	16.53	15.78	15.77	16.06	16.05	16.83
Penta-	2.24	2.01	3.26	3.75	3.36	2.93	4.10	3.42	3.52	4.34
Hexa	0.25	0.38	0.54	0.62	0.54	0.42	0.52	0.52	0.52	0.66
Total	100.00	100.00	100.00	100.00	100.00	100.00	100.00	100.00	100.00	100.00

Supplementary Table 7. The AT-content in the 5'UTRs, CDSs, introns, 3'UTRs,TEs, and intergenic regions of the primates genome

Regions	*OtoGar*	*CalJac*	*MacMul*	*ChlSab*	*PapAnu*	*NomLeu*	*GorGor*	*PonAbe*	*PanTro*	*HomSap*
5'UTRs	45.61	43.24	43.23	41.91	41.15	42.45	41.14	39.81	39.56	42.30
CDSs	48.48	48.45	48.47	48.00	47.88	48.51	47.87	48.00	48.07	47.86
3'UTRs	58.46	58.03	56.68	56.19	53.63	57.44	56.85	56.83	58.23	56.02
TEs	58.60	58.40	58.22	58.20	58.23	58.20	58.54	58.48	58.36	58.28
Introns	58.61	58.50	58.79	58.28	58.39	58.57	59.16	58.66	58.60	58.64
Intergenic regions	59.18	59.83	59.74	59.81	59.71	60.15	60.09	59.77	59.73	59.93

Supplementary Table 8. The AT-content (%) of mono- to hexanucleotide P-SSRs in the 5'UTRs of the primate genomes

Type	*OtoGar*	*CalJac*	*MacMul*	*ChlSab*	*PapAnu*	*NomLeu*	*GorGor*	*PonAbe*	*PanTro*	*HomSap*
Mono-	96.02	93.27	96.42	95.07	96.84	96.18	95.36	93.52	94.47	96.49
Di-	43.33	46.71	46.99	46.83	47.25	46.81	47.94	46.56	45.74	48.45
Tri-	12.53	12.58	12.40	12.28	10.43	12.32	12.20	10.63	7.92	11.12
Tetra-	28.77	30.99	32.48	42.36	46.34	38.19	26.39	44.78	27.40	38.62
Penta-	20.00	20.79	30.83	41.13	28.02	30.15	19.56	26.16	26.03	29.67
Hexa-	16.67	12.62	31.79	33.33	27.88	25.25	18.52	14.80	19.68	23.86
Total	26.63	32.35	37.73	49.66	42.90	46.84	32.78	40.63	29.18	33.67

[a] The numbers of nucleotides in SSRs are listed. For example: the total of the nucleotides in mononucleotide SSRs are 5458214 bp, one of which have 5316524 bp A+T and 141690 bp C+G.
[b] The percentage of nucleotides are shown in table.

Supplementary Table 9. The AT-content (%) of mono- to hexanucleotide P-SSRs in the CDSs of the primate genomes

Type	*OtoGar*	*CalJac*	*MacMul*	*ChlSab*	*PapAnu*	*NomLeu*	*GorGor*	*PonAbe*	*PanTro*	*HomSap*
Mono-	98.58	94.42	98.44	97.96	97.78	94.36	94.20	95.56	94.06	94.72
Di-	61.21	50.04	53.25	52.99	50.00	50.08	51.69	52.87	48.48	49.07
Tri-	33.50	33.41	31.69	29.85	31.33	31.35	33.07	30.49	29.45	30.89
Tetra-	65.58	57.18	65.87	60.23	53.04	48.67	61.91	50.91	53.27	49.51
Penta-	80.00	60.00	72.73	64.84	60.00	58.83	59.07	48.67	75.79	55.85
Hexa-	28.82	29.31	30.55	29.51	24.37	34.45	32.32	27.35	29.24	31.77
Total	42.19	37.74	45.40	35.99	33.05	42.59	41.21	35.18	31.21	32.08

Supplementary Table 10. The AT-content (%) of mono- to hexanucleotide P-SSRs in the introns of the primate genomes

Type	OtoGar	CalJac	MacMul	ChlSab	PapAnu	NomLeu	GorGor	PonAbe	PanTro	HomSap
Mono-	97.96	99.68	98.66	99.44	99.71	98.71	99.63	99.37	99.43	99.42
Di-	55.09	56.98	58.21	59.01	59.46	59.62	60.92	61.56	58.21	62.51
Tri-	63.91	64.5	71.33	73.11	66.3	70.2	68.22	70.18	68.17	69.31
Tetra-	77.07	72.04	79.38	73.19	74.61	86.02	79.89	78.61	80.8	80.17
Penta-	72	75.48	81.37	80.56	73.01	86.15	80.41	73.25	73.33	70.73
Hexa-	81.67	84.42	77.54	78.21	81.33	81.33	81.33	80.63	83.33	83.33
Total	88.72	85.1	87.13	88.2	89.25	87.19	88.31	88.72	88.91	89.28

Supplementary Table 11. The AT-content (%) of mono- to hexanucleotide P-SSRs in the 3'UTRs of the primate genomes

type	*OtoGar*	*CalJac*	*MacMul*	*ChlSab*	*PapAnu*	*NomLeu*	*GorGor*	*PonAbe*	*PanTro*	*HomSap*
Mono-	98.36	99.01	99.47	98.85	99.28	99.26	98.32	99.15	98.17	98.95
Di-	64.22	62.19	60.59	57.66	57.86	60.02	59.68	58.17	59.89	60.60
Tri-	50.21	55.46	57.51	65.26	64.57	64.96	62.13	63.69	62.84	58.47
Tetra-	68.64	63.37	65.77	70.40	74.95	73.52	69.47	71.82	70.76	74.48
Penta-	67.69	58.83	67.69	75.56	76.11	72.59	70.26	71.84	72.42	73.19
Hexa-	45.00	42.59	50.00	64.45	55.02	47.99	60.67	58.06	62.06	71.55
Total	86.83	82.33	84.02	86.70	87.90	86.86	82.17	86.53	83.91	83.50

Supplementary Table 12. The AT-content (%) of mono- to hexanucleotide P-SSRs in the TEs of the primate genomes

Type	*OtoGar*	*CalJac*	*MacMul*	*ChlSab*	*PapAnu*	*NomLeu*	*GorGor*	*PonAbe*	*PanTro*	*HomSap*
Mono-	99.56	99.81	99.67	99.67	99.67	99.86	99.74	99.82	99.76	99.86
Di-	62.94	63.24	61.33	62.33	58.33	64.41	63.82	63.95	63.72	64.76
Tri-	69.10	74.01	72.86	74.86	73.86	75.90	74.89	76.74	75.04	75.33
Tetra-	84.74	81.42	82.85	82.85	84.85	85.67	84.66	85.64	85.16	85.20
Penta-	79.35	81.08	80.16	82.60	83.60	83.08	82.44	83.15	82.91	82.64
Hexa-	73.44	78.11	78.30	79.30	80.30	83.10	82.75	83.89	84.69	82.59
Total	95.08	95.35	94.67	95.49	96.05	96.18	94.88	95.78	95.56	95.88

Supplementary Table 13. The AT-content (%) of mono- to hexanucleotide P-SSRs in the intergenic regions of the primate genomes

Type	OtoGar	CalJac	MacMul	ChlSab	PapAnu	NomLeu	GorGor	PonAbe	PanTro	HomSap
Mono-	98.69	99.32	99.65	99.43	99.56	99.64	99.63	99.42	99.13	99.62
Di-	60.67	59.45	58.37	57.00	57.90	61.96	59.80	60.69	61.59	61.99
Tri-	70.33	69.04	73.01	72.01	72.48	74.18	73.67	78.29	73.69	74.06
Tetra-	76.57	69.65	73.00	72.11	72.37	73.56	75.84	76.07	75.59	74.18
Penta-	74.73	69.28	79.80	78.71	78.73	77.96	73.16	79.44	78.56	74.12
Hexa-	65.26	62.37	71.12	72.09	69.03	69.66	67.03	68.32	71.12	64.18
Total	86.82	79.83	83.56	82.59	83.51	83.69	88.36	84.61	83.96	82.65
